# Seropositivity of Dengue Antibodies during Pregnancy

**DOI:** 10.1155/2014/436975

**Published:** 2014-12-22

**Authors:** Nor Azlin Mohamed Ismail, Wan Elly Rushima Wan Abd Rahim, Sharifah Azura Salleh, Hui-Min Neoh, Rahman Jamal, Muhammad Abdul Jamil

**Affiliations:** ^1^Department of Obstetrics & Gynaecology, Medical Faculty, Universiti Kebangsaan Malaysia, Jalan Yaacob Latiff, 56000 Cheras, Kuala Lumpur, Malaysia; ^2^Department of Microbiology, Medical Faculty, Universiti Kebangsaan Malaysia, Jalan Yaacob Latiff, 56000 Cheras, Kuala Lumpur, Malaysia; ^3^UKM Medical Molecular Biology Institute, Medical Faculty, Universiti Kebangsaan Malaysia, Jalan Yaacob Latiff, 56000 Cheras, Kuala Lumpur, Malaysia

## Abstract

*Purpose*. Malaysia a dengue endemic country with dengue infections in pregnancy on the rise. The present study was aimed at determining dengue seroprevalence (IgG or IgM) during pregnancy and its neonatal transmission in dengue seropositive women. *Methods*. Maternal with paired cord blood samples were tested for dengue antibodies (IgG and IgM) using an enzyme-linked immunosorbent assay (ELISA). Maternal age, parity, occupation, ethnic group, and gestational age were recorded. Data on neonatal Apgar score and admissions to the Neonatal Intensive Care Unit (NICU) were analyzed. *Results*. Out of 358 women recruited, about 128 (35.8%) patients were seropositive. Twelve patients (3.4%) had recent infections (IgM positive) and another 116 women (32.4%) were with past infections (IgG positive). All babies born to seropositive mothers had positive IgG paired cord blood; however, no IgM seropositivity was observed. All neonates had good Apgar scores and did not require NICU admission. *Conclusion*. In this study, 35.8% pregnant women were found to be dengue seropositive. However, transplacental transfer of IgG antibodies had no detrimental effect on the neonatal outcomes.

## 1. Introduction

Dengue is spread through daytime-biting* Aedes aegypti* mosquitoes which release RNA viruses (flavivirus). Although four different antigenic varieties of dengue viruses are recognized, infection with one serotype does not confer immunity to the other three. As described, the first encounter of dengue fever (primary dengue) predisposes a person to more severe manifestations of the disease after infection with other serotypes (secondary dengue) [1]. Being an endemic country, Malaysia has all four dengue virus serotypes present in local circulation [2].

Perret et al. [3] found a dengue seropositivity rate of 94.7% in a highly endemic area in Thailand. Dengue seropositivity rates increase with advance maternal age. As a result, older patients are more likely to have preexisting protective immunity [3] whilst younger women are more at risk to contract primary disease during pregnancy.

In Malaysia, reported cases of clinical dengue have increased drastically to 42,140 cases of dengue fever (DF) with an incidence rate of 148.73 per 100,000 population; while for dengue haemorrhage fever (DHF), a total of 41,031 cases (14.23 per 100,000 population) were reported in the year 2010 [4]. An increase of up to 63% of dengue antibodies was detected in those aged 21 to 40 years in a nonpregnant suburban community in Malaysia [5]. Therefore, the infection rate of either primary or secondary dengue would overall be relatively higher during pregnancy. Dengue in pregnancy is known to cause complications involving maternal mortality, low birth weights, preterm delivery, neonatal admission, and fetal death [6, 7].

The vertical transmission of dengue infections with detection of IgM in cord serum has been infrequently reported, although isolated reports from Cuba, Brazil, and Thailand during outbreaks have been noted [8]. A spectrum of neonatal outcomes range from asymptomatic infection to death is identified. Dengue infection is known to cause health complications to newborns of infected mothers, even in asymptomatic maternal infection. The most frequently used serological tests for dengue are the haemagglutination inhibition (HI) assay and IgG or IgM enzyme immunoassays. ELISA is one of the most commonly used tests for rapid confirmation of dengue infections [9]. This present study was aimed at determining maternal dengue seroprevalence (IgG or IgM) of dengue infection during pregnancy and the neonatal transmission in women who were dengue seropositive.

## 2. Material and Methods

This was a cross-sectional study conducted over a period of five months at a teaching hospital. All women admitted for delivery during the study duration were briefed about the study and invited to participate. For consenting participants, informed and written consent was obtained in the delivery room once the patient had been admitted in early phase of active labour. Foreigners and those with multiple pregnancies were excluded from this study.

### 2.1. Biochemical Analysis

Ten milliliters of maternal blood and five milliliters of cord blood sample were collected and tested for dengue IgM and IgG using the same ELISA kit. Briefly, after blood was collected, the samples were centrifuged and the sera were kept at −20°C. Each ELISA test contains a microplate, which was precoated with mouse monoclonal anti-human IgM or IgG antibodies in wells. During first incubation with the microplate, anti-dengue IgM or IgG antibody in patient's serum will first bind to mouse monoclonal anti-human IgM or IgG antibodies coated wells and subsequently to the mixture of dengue antigen and mouse monoclonal anti-dengue conjugate. Following this, all unbounded materials were removed by aspiration and washing. The remaining enzyme activity found in the wells was directly proportional to the dengue IgM or IgG antibody concentration in patient's serum and was evidenced by incubating the solid-phase with a substrate solution in a substrate buffer. A spectrophotometer at 450 nm was used to perform colorimetric reading.

Maternal age, parity, current address, occupation, gestational age, and ethnic group were recorded. Data on neonatal Apgar score and admissions to the Neonatal Intensive Care Unit (NICU) were collected. These data were analysed from the delivery records. All collected data was analysed using the statistical package SPSS. Descriptive statistics are shown as mean ± standard deviation. The Chi-square test was used for categorical comparison for each demographics. Values of *P* less than or equal to 0.05 were considered as statistically significant.

## 3. Results

A total of 358 women who all delivered via spontaneous vaginal delivery were recruited. Among these, the youngest and the oldest were 19 years and 41 years, respectively. Mean age was 28.89 ± 4.43-year old. Majority (60.3%) of the patients were between 21 and 30 years. Malays were the majority (60%) ethnic group. Slightly more than half of the patients (55%) were multiparae. About 227 (63.4%) of the studied subjects were working and 131 (36.6%) women were housewives. Mean gestational age was 38.86 ± 1.19 weeks with majority (81%) of them at term.

A total of 128 patients (35.8%) were dengue seropositive (Table 1). From this study, majority of dengue seropositive patients were in the 21–30 age group (53%), were Malay (82%), were having parity 2 to 4 (62%), and were housewives (54%). There were more seropositivity rates at term (Table 2). All babies born to seropositive mothers had paired cord samples with positive dengue IgG; however, none of them were IgM positive. The neonates had good Apgar scores of more than 7 in one minute and all babies did not require NICU admission.

## 4. Discussion

Antibodies against dengue are broadly reactive with other flaviviruses. Therefore, a specific accurate diagnostic method for acute dengue virus infection is of great importance. A local study [10] reported dengue infections in pregnancy (dengue-specific IgM positive) in about 2.5% of its participants in a Malaysian setting. A relatively low vertical transmission of 1.6% by only one fetus with no neonatal manifestation at birth was reported. This was also observed in the present study where all the neonates with seropositive mothers had positive IgG; however, none showed positive IgM and had remained well. This is probably explained by a lower (35.8%) dengue seropositivity rate in our population of pregnant women compared to other endemic countries such as Thailand which reported a seroprevalence of 94.7% dengue in the year 2005. Another local retrospective study [6] had reported only 16 cases of dengue in pregnancy between 2000 and 2004 with 50% dengue IgM positive cases while others had only positive Tourniquet test during the diagnosis.

The DHF risk in neonates is associated strongly with primary infection, which is also related to passively acquired maternal antibodies. During the first 6 months of life when antibody titres are high, maternal antibodies protect infant from dengue infection. However, in older infants and children, it has been reported that a higher risk of DHF was found together with decrease in titres of transplacentally acquired antibodies [11].

Primary dengue infection may present with mild to high fever, myalgia, headache, and skin rashes. IgM antibodies produced by fifth day of the symptoms may last for 30 to 180 days. IgG appears on the 14th day and persists for life. In secondary infections, besides high fever, hemorrhagic events and circulatory failure may occur. It has been shown that dengue IgG rises within 1 to 2 days of the symptoms and induces IgM response following 20 days of infection [12]. In our study, 116 patients (32.4%) were detected to be IgG positive while only 12 patients (3.4%) had positive IgM.

Perret et al. in 2005 [3] had identified maternal age as the only risk factor related to dengue infection in pregnancy as mothers in the older age group (>20 years) were significantly more likely to be seropositive than younger women. This finding was similar to the present study as a larger number of older patients (>20 years) were found to have higher seropositivity. There were 8 out of 12 patients (66.7%) who were IgM positive in this age group. Age factor has linear correlations with parity [7]. Similar to past studies, our present study also showed that majority of our study subjects were in their third trimester of pregnancy [7].

Majority of our study population belonged to the Malay ethnic group which was similar to those staying around our centre catchment area. Among those detected to be IgM positive, the highest were the Malays, 11 patients (91.7%). Interestingly, 8 out of 12 patients (66.7%) who were dengue IgM positive were housewives; they might have been exposed at their own home. The community or neighbourhood where majority of the ethnic group was Malay might have the same sanitary and drainage system which promote breeding site for the* Aedes aegypti* mosquito. Malaysia as one of the endemic countries for dengue needs larger scale epidemiology studies in looking at areas of residences before appropriate actions could be taken in preventing the spread of dengue infections.

In the present study, out of 12 patients who were detected with positive IgM, only six women (50%) had a febrile illness in pregnancy, either during first, second, or third trimester. Through a follow-up phone call, these patients had complaints of having fever with nonspecific symptoms of headache and myalgia. Only one patient was diagnosed to be in day 9 of dengue fever during labour. Study in a similar setting [10] that noted 88.9% of those with dengue IgM also did not experience febrile illness during their pregnancy. In countries with dengue endemicity high frequency of anti-dengue antibodies were reported in maternal blood [13], which is also the case in the present study. Majority of the cases were treated conservatively as in other parts of the world [14].

Adverse pregnancy outcomes reported in seropositive mothers with maternal clinical dengue infection involving maternal mortality, preterm delivery, fetal anomalies, low birth weight, and miscarriage [6, 7] were not seen in this present study. The limitation in this study was that the result of seropositive samples could have been confirmed by a supplemental assay such as RT-PCR and HI (Hemagglutination Inhibition) tests as the gold standard [9]. Unfortunately, RT-PCR and HI tests were not available at our centre due to lack of antigen that had to be specifically ordered from our neighbouring dengue endemic country, Thailand. These additional tests would have provided some additional information such as dengue serotypes and quantification of past or recent dengue infections while correlating this to the neonatal outcomes.

## 5. Conclusion

In our study, 35.8% pregnant women were found to be dengue seropositive. However, transplacental transfer of IgG antibodies had no detrimental effect on the neonatal outcome.

## Figures and Tables

**Table 1:** Seroprevalence status.

Antibodies	*n*	%
Recent infection (IgM)	12	3.4
Past infection (IgG)	116	32.4
Seronegative	230	64.2

Total	358	100

**Table 2:** Seroprevalence versus maternal features.

	Seronegative		Seropositive		*P* value
	*n*	%	*n*	%	
Age					
16–20	11	5	2	2	**0.013**
21–30	148	64	68	53	**<0.001**
31–40	68	30	58	45	0.373
>40	3	1	0	0	—
Race					
Malay	169	73	105	82	**<0.001**
Chinese	52	23	19	15	**<0.001**
Indian	9	4	4	3	0.166
Parity					
1	108	47	39	30	**<0.001**
2–4	118	51	79	62	**0.005**
5–9	4	2	10	8	0.109
Gestation age					
<36 + 6 weeks	9	4	5	4	0.285
37-37 + 6 weeks	33	14	20	16	0.074
38–40 + 9 weeks	188	82	103	80	**<0.001**
Occupation					
Housewife	62	27	69	54	0.541
Working	168	73	59	46	**<0.001**

*P*  values <0.05 were accepted as significant.
